# Effect of Biochar on Metal Distribution and Microbiome Dynamic of a Phytostabilized Metalloid-Contaminated Soil Following Freeze–Thaw Cycles

**DOI:** 10.3390/ma15113801

**Published:** 2022-05-26

**Authors:** Maja Radziemska, Mariusz Z. Gusiatin, Agnieszka Cydzik-Kwiatkowska, Aurelia Blazejczyk, Vinod Kumar, Antonin Kintl, Martin Brtnicky

**Affiliations:** 1 Institute of Environmental Engineering, Warsaw University of Life Sciences, Nowoursynowska 159, 02-776 Warsaw, Poland; 2 Faculty of Geoengineering, University of Warmia and Mazury in Olsztyn, Słoneczna St. 45G, 10-719 Olsztyn, Poland; mariusz.gusiatin@uwm.edu.pl (M.Z.G.); agnieszka.cydzik@uwm.edu.pl (A.C.-K.); 3 Institute of Civil Engineering, Warsaw University of Life Sciences, Nowoursynowska 159, 02-776 Warsaw, Poland; aurelia_blazejczyk@sggw.edu.pl; 4 Department of Botany, Government Degree College, Ramban 182144, India; vinodverma507@gmail.com; 5 Department of Agrochemistry, Soil Science, Microbiology and Plant Nutrition, Faculty of AgriSciences, Mendel University in Brno, Zemedelska 1, 61300 Brno, Czech Republic; antonin.kintl@mendelu.cz (A.K.); martin.brtnicky@mendelu.cz (M.B.); 6 Agricultural Research, Ltd., Zahradni 400/1, 66441 Troubsko, Czech Republic; 7 Institute of Chemistry and Technology of Environmental Protection, Faculty of Chemistry, Brno University of Technology, Purkynova 118, 61200 Brno, Czech Republic

**Keywords:** phytoremediation, soil carbon-type amendments, biochar, soil freeze–thaw, post-industrial urban areas

## Abstract

In the present paper the effectiveness of biochar-aided phytostabilization of metal/metalloid-contaminated soil under freezing–thawing conditions and using the metal tolerating test plant *Lolium perenne* L. is comprehensively studied. The vegetative experiment consisted of plants cultivated for over 52 days with no exposure to freezing–thawing in a glass greenhouse, followed by 64 days under freezing–thawing in a temperature-controlled apparatus and was carried out in initial soil derived from a post-industrial urban area, characterized by the higher total content of Zn, Pb, Cu, Cr, As and Hg than the limit values included in the classification provided by the Regulation of the Polish Ministry of Environment. According to the substance priority list published by the Toxic Substances and Disease Registry Agency, As, Pb, and Hg are also indicated as being among the top three most hazardous substances. The initial soil was modified by biochar obtained from willow chips. The freeze–thaw effect on the total content of metals/metalloids (metal(-loid)s) in plant materials (roots and above-ground parts) and in phytostabilized soils (non- and biochar-amended) as well as on metal(-loid) concentration distribution/redistribution between four BCR (community bureau of reference) fractions extracted from phytostabilized soils was determined. Based on metal(-loid)s redistribution in phytostabilized soils, their stability was evaluated using the reduced partition index (Ir). Special attention was paid to investigating soil microbial composition. In both cases, before and after freezing–thawing, biochar increased plant biomass, soil pH value, and metal(-loid)s accumulation in roots, and decreased metal(-loid)s accumulation in stems and total content in the soil, respectively, as compared to the corresponding non-amended series (before and after freezing–thawing, respectively). In particular, in the phytostabilized biochar-amended series after freezing–thawing, the recorded total content of Zn, Cu, Pb, and As in roots substantially increased as well as the Hg, Cu, Cr, and Zn in the soil was significantly reduced as compared to the corresponding non-amended series after freezing–thawing. Moreover, exposure to freezing–thawing itself caused redistribution of examined metal(-loid)s from mobile and/or potentially mobile into the most stable fraction, but this transformation was favored by biochar presence, especially for Cu, Pb, Cr, and Hg. While freezing–thawing greatly affected soil microbiome composition, biochar reduced the freeze–thaw adverse effect on bacterial diversity and helped preserve bacterial groups important for efficient soil nutrient conversion. In biochar-amended soil exposed to freezing–thawing, psychrotolerant and trace element-resistant genera such as *Rhodococcus* sp. or *Williamsia* sp. were most abundant.

## 1. Introduction

One of the negative effects of the progress of civilization is the increasing contamination of individual components of the natural environment resulting from the interaction of three main groups of factors—physical, chemical, and biological—which are directly connected with human activity [1]. Among the threats to the soil environment, there are various types of chemical inorganic contaminants, which include metals/metalloids (abbr. metal(-loid)s) [2]. The forms in which metal(-loid)s are found in soil are one of the main chemical factors determining their mobility in the natural environment as well as the level of their toxicity [3]. It is the bioavailable forms that are easily taken up by living organisms and moved along the sequential links of the food chain, thus posing the greatest threat [4]. Moreover, metal(-loid)s that cause soil contamination obstruct the development of the microorganisms found within, which leads to a disturbance in processes connected with decomposition and transformation of organic matter [5]. A disturbance of the decomposition of organic matter by a microorganism may also lead to increased bioavailable forms of metals in the soil.

Over 10 million contaminated areas have been identified throughout the world, of which over half are contaminated with metal(-loid)s [6]. Studies on the scale of human-induced environmental changes, the determination of their influence on the environment as well as the development of new and effective means of remediation are increasingly important directions of scientific research throughout the world [7]. Various kinds of post-industrial urban areas that are highly contaminated by metal(-loid)s merit particular attention. The soils derived from these areas contain large amounts of potentially toxic chemical elements, among which As, Pb, Hg (herein under study), and Cd are currently indicated as being among the top 10 most hazardous substances [8,9]. These strategic areas that are localized in cities pose a scientific challenge due to the aspirations and necessity of reusing them as, inter alia, public areas that can be used by local people as an informal recreational areas. Introducing modern remediation technologies may significantly limit the pressure imposed on the soil environment. In recent years, nature-based solutions applied to remediate areas contaminated with metal(-loid)s, which most certainly includes the technique of aided phytostabilization, have been taking on increasing importance [10]. This technique relies on using the ability of specific plants to produce a good amount of biomass as well as to develop a dense and strong root system that mostly accumulates contaminants and, by doing so, immobilizes them in soil. At the same time, the above-ground part of plants is expected to be contamination-free and thus will not pose a threat to living organisms [11]. Maintaining a ‘permanent’ plant cover on contaminated land also limits the risk of exposure to soil contact and controls surface erosion, as well as preventing contamination spread from the weathered zone to the surrounding area (dust emission) and further runoff down to the valleys in the event of precipitation [12]. Processes, which take place during phytostabilization, can be aided by the addition of appropriate immobilizing amendments to soil [13]. In recent years, various kinds of materials have been commonly used to immobilize metal(-loid)s in soils, largely due to their availability and low costs. Among the large assortment of amendments, a lot of focus has been placed on the use of biochar obtained from an original biomass feedstock [14]. The list of areas in which biochar can be applied is constantly increasing, though the material is currently being used mainly in agriculture and the widely understood field of environmental engineering and protection. One of the directions of using biochar is its use as a soil amendment in the aided phytostabilization technique [15]. Its application raises soil pH value, and increases the effect of immobilizing the metal(-loid)s mobile forms (from labile to less-labile) by different processes such as adsorption (which includes complexation by functional groups of amendments or attracting electrostatic interactions to a charged surface of amendments), co-/precipitation, cation-exchange (available negatively charged sites on the soil surface) and reduction of elements, thus decreasing their overall bioavailability and toxicity for plants, as well as increasing the amount of organic carbon and ability to retain water in soil [16].

Two of the most important physical factors which have an influence on the processes taking place in the natural soil environment are its temperature and water content; at the same time, they are among the factors that are most variable over time on a fixed local scale. Changes in the temperature of the surroundings are an important factor influencing soil-forming processes [17]. They determine, among others, soil colloidal properties, the rate of chemical reactions, and the activity of the soil microbiome, as well as the freezing and thawing processes of water in soil [18]. Moreover, low temperature may lead to a series of unfavorable physiological, metabolic and structural changes in plants, which consequently lead to damage to plant cells [19]. Freeze–thaw (FT) conditions/cycles, by changing the physical and chemical properties of soil [20], influence the transformation of metal(-loid)s in an integrated soil and plant system. Many studies conducted so far focus solely on the changes of selected physical (including mechanical) properties of soil (with and without amendments) during freeze–thaw conditions [21,22]. However, there is no data available on the effect of freeze–thaw on an integrated system, such as soil–amendment–plant, in which the sorption properties and, in consequence, the metal(-loid)s mobility versus immobility in soil and bioaccumulation in plants, are taken into account. Hence, the authors of the present paper touched on a very important issue which reveals the behavior of metal(-loid)s, both in soil and in plants, following freeze–thaw treatment, and at the same time answering the question of whether aided phytostabilization, (i.e., in an integrated experimental setup, soil with an amendment and plant cover) occurs at equal efficiency under such conditions as at a moderate temperature (16 °C to 26 °C). To the authors’ knowledge, this strategy had not been used to date.

Revealing connections between the metal-accumulating plants and the microbiome that inhabits the rhizosphere (soil region in the vicinity of plant roots) is crucial to implementing successful phytoremediation [23]. The low activity of microorganisms in the rhizosphere is the main biological factor obstructing the growth of plants and negatively affecting their resistance (tolerance) to pathogens [1].

In the above context, the aim of the present paper was to determine (1) the amount of metal(-loid)s (Zn, Pb, Cu, Cr, As, Hg) occurring in soil; (2) the level of metal(-loid)s accumulating in plant tissues (roots and above-ground parts); (3) the stability of metal(-loid)s using the reduced partition index (Ir) by taking into account the metal(-loid)s redistribution in soil; (4) the soil microbial composition following phytostabilization (non- and biochar-amended), in combination with no exposure to freeze–thaw (glass greenhouse temperatures) and following exposure of soil cultivated with plants to multiple freeze and thaw (FT-chamber temperatures; Toropol K-010 apparatus). In each freeze–thaw cycle, soil with plants (enclosed in FT-chamber) was first kept at an air temperature of −20 ± 0.5 °C for 48 h and thawed at 20 ± 0.5 °C for 48 h, maintaining air pressure at the level of 1000 ± 10 hPa. Experimental data were collected for phytostabilization conducted under the following conditions: (1) greenhouse temperatures (before freeze–thaw), up to 52 days (d) and (2) greenhouse temperatures, followed by FT chamber temperatures (after freeze–thaw treatment), up to a total of 116 d.

## 2. Materials and Methods

### 2.1. Characteristics of Initial Soil, Biochar, and Plant

Surface soil (0–0.25 m) was taken from an area in central Poland where steel and metal waste had been stored directly on the ground for many years. About 70 kg of representative soil was collected by taking 7 sub-samples (each 10 kg) from 1 × 1 square meter quadrants. The sampled soil was transferred to appropriately marked polyethylene (PE) bags and transported to the laboratory, where all sub-samples were combined and mixed to form a single bulk soil material and pre-dried at room temperature for 72 h and sifted using 2 mm mesh to (1) separate a small average laboratory sample to characterize initial soil and (2) prepare initial soil for the vegetative experiment involving plant growth, its above-ground parts and roots, before (in greenhouse) and after multiple freeze–thaw treatments (in a greenhouse, followed by the FT chamber). Based on particle size distribution, the initial soil was classified as loamy sand (71.6% sand, 27.2% silt, 1.2% clay). It was characterized by low moisture content immediately after delivery to the laboratory, excluding pre-drying at room temperature (9.5%, *w*/*w*), organic matter (OM) content (1.11 ± 0.10%), and an alkaline pH value (8.7 ± 0.16). Moreover, the metal(-loid)s content in mg/kg of initial soil was as follows: Zn 9129 ± 5.58, Pb 2132 ± 13.44, Cu 1278 ± 5.59, Cr 637 ± 6.99, As 393 ± 3.11 and Hg 62.03 ± 1.16 (based on AAS measurements), and were higher than the permissible levels included in the classification provided by the Regulation of the Polish Ministry of Environment [24]. Currently, the accepted limit values of contaminants tested in this study in mg/kg of soil are as follows: Zn 1000, Pb 600, Cu 600, Cr 500, As 60 and Hg 30. The rest of sieved initial soil was placed in PE bags and stored in a refrigerator at temperature of 4 °C until the time of setting up the vegetative experiment in a glass greenhouse.

Biochar was produced by Fluid S.A. company (Sędziszów, Poland). The raw material comprised willow chips, and biochar was made through thermolysis (thermal treatment) of a biomass feedstock at 650 °C for 15 min at a heating rate of about 3 °C/s, under no oxygen conditions [25]. Prior to the vegetative experiment, biochar was specially milled and sifted to obtain a particle size less than or equal to 0.5 mm. It was characterized by an alkaline pH value (10.2 ± 0.3) and a surface area (313.73 m^2^/g). The metal(-loid)s content in mg/kg of biochar was as follows: Zn 200.2 ± 10.7, Cr 9.6 ± 0.7, Cu 3.9 ± 1.3, As 1.8 ± 1.2, Pb 1.1 ± 1.6 and Hg 0.06 ± 1.1 (based on AAS measurements). Moreover, the elemental composition of biochar particles not-exposed to freeze–thaw conditions was as follows (%, *w*/*w*): C 63.2 ± 7.4, O 20.4 ± 1.3, Ca 9.78 ± 4.7, K 2.6 ± 0.8, Cu 2.0 ± 0.7, P 0.8 ± 0.6, Mg 0.43 ± 0.38, Al 0.39 ± 0.17, Mn 0.15 ± 0.15, Fe 0.12 ± 0.07, S 0.11 ± 0.07, Si 0.06 ± 0.03 (based on SEM-EDS measurement). The milled biochar (alone) was additionally subjected to the same freeze–thaw procedure as pots containing contaminated soil cultivated with plants following the carried out (non- and biochar-amended) phytostabilization, to reliably assess the freeze–thaw impact on the changes of selected physical properties of biochar such as specific surface area, surface morphology and ‘atypical cracks’ (Section 3.2).

*Lolium perenne* L., a perennial ryegrass, as a plant species selected for this study, in natural environmental conditions is best adapted to areas with a mild climate, its optimum growth temperature is 18–20 °C [26]. Although this grass species is cold tolerance, it may not survive very cold winters (−6 °C or lower) [27]. It can tolerate both acidic and alkaline soils, with pH ranging from 5.1 to 8.4 and its best growth occurs when the soil pH ranges from 5.5 and 7.5 [28]. It is recommended as the most appropriate species for revitalization of soils contaminated with metal(-loid)s from metallurgical sites, metalliferous mineral wastes, and mine tailings [29,30,31]. Moreover, this grass species does not present mechanisms of cation hyperaccumulation in its above-ground parts under field conditions (in amounts greater than those found in the soil) during cation uptake and is recommended as a valuable tool for bioavailability assessment [32,33,34]. It is characterized by a fast growth rate, both in the sowing year and in the following years, as well as a high yielding potential.

### 2.2. Soil Biochar-Aided Phytostabilization (before and after Freeze-Thaw Treatment, Respectively)

The vegetative experiment was started in a glass greenhouse. *Lolium perenne* L. certified seeds produced by Olznas Sp. z o.o. (Authorized Seed Production Centre, Olsztyn, Poland) were sown in the amount of 5 g per 5.0 kg soil per pot (with day 0 being the date of sowing). A total of six out of twelve pots (in total) were filled with a mix of initial soil and biochar (3.0%, *w*/*w*) to obtain biochar-amended soil and the remaining six with initial soil without biochar (0.0%, *w*/*w*) denoted as non-amended (control) soil. Prior to sowing, all prepared soil mixes were placed in pots and covered with Al-foil on the top to avoid light penetration and conditioned for over two weeks to stabilize under controlled laboratory conditions at an air temperature of 20 ± 2 °C, relative air humidity (RH) of 50 ± 10%, and air pressure of 1000 ± 10 hPa. Next, just before sowing, soil mixes (biochar 0% and 3%, *w*/*w*) were adjusted to 60% of their maximum water holding capacity. Pots (14 cm in diameter and 18 cm in height) containing no drainage holes were used to avoid leaching of contaminants from soil mixes. Typically, grass seeds take 5 to 10 d to germinate; hence, tops of the pots were covered with transparent foil in the first 5 d after sowing to prevent water loss, and the incubated. *Lolium perenne* L. cultivation period in twelve pots was 52 d (1248 h) before freeze–thaw treatment under stable greenhouse conditions, at a temperature of 26 ± 3 °C during the day (14 h) and 16 ± 2 °C at night (10 h), an RH of 60 ± 10%, and with irrigation two or three times per week to 60% of the maximum water holding capacity of soil. No fertilizer was applied to assist plant growth in order to avoid interactions with biochar. Plants were harvested 52 d after sowing from only three biochar-amended and three control pots, thus finishing the greenhouse cultivation stage, whereas the not-harvested plants in the remaining three biochar-amended and three control pots were placed in a temperature-controlled chamber (Toropol K-010 apparatus, 288 L in working volume, Warsaw, Poland) for a period of 64 d (1536 h) to examine the freeze–thaw effect on biochar-aided phytostabilization. For this purpose, an experimental procedure elaborated by Hou et al. [35] was used, originally applied in the soil-amendment (various) system to determine the freeze–thaw impact on metal mobility in (Cd, Pb)-contaminated soil. Herein, an analogous set of parameters and values, such as temperature, time, and number of cycles were implemented in the extended soil–amendment–plant system. A total of 16 freeze-thaw cycles were run in a closed FT chamber. In each freeze–thaw cycle, air temperature was cycled between −20 ± 0.5 °C, RH of 90 ± 10%, for 48 h (freezing) followed by 20 ± 0.5 °C, an RH of 50 ± 10%, for 48 h (thawing), with air pressure at 1000 ± 10 hPa.

### 2.3. Analytical and Other Methods

Following phytostabilization (non- and biochar-amended) carried out before and after freeze–thaw treatment, the above-ground parts and roots of *Lolium perenne* L. and, phytostabilized soils were separated and sorted from each pot. The above-ground fresh biomass was weighted using a precision balance (Acculab ATL-623-V, Sartorius, Goettingen, Germany). The roots were carefully and quickly washed under running tap water from soil aggregates and afterward with deionized water, and net blotted dry with absorbent paper for a few seconds. Next, the individual parts of plants (stems and roots) were dried at room temperature and, finally, oven-dried at 55 °C to a stable mass, with the dry biomass recorded separately for each pot. The phytostabilized soils were ground to obtain a more homogeneous soil material, separately for each pot, using a soil grinder (H-4199.5F, Humboldt Mfg. Co., Elgin, IL, USA) and dried only at room temperature. In order to determine the metal(-loid) content by atomic absorption spectrometry (AAS), powdered plant samples were prepared from each part of the plant using a cutting mill (SM 100, Retsch, Hann, Germany), whereas phytostabilized soil samples were additionally sifted using 1 mm mesh. The powdered plant materials were digested in a mixture of 65% HNO_3_ and 30% H_2_O_2_ using a microwave oven (MARSXpress, CEM Corporation, Matthews, NC, USA). The soil materials (phytostabilized soils and also initial soil) were digested in a mixture of 36% HCl, 65% HNO_3,_ and 30% H_2_O_2_. All chemicals were of analytical reagent grade. During filtration, the digested material solutions (plant or soil) were filled with ultrapure water to a flask volume of 100 mL (Milli-Q System, Merck Millipore, Merck KGaA, Darmstadt, Germany). Finally, the diluted solutions of digested materials were analyzed for Zn, Pb, Cu, Cr, and As levels by a flame atomic absorption spectrometry (FAAS) using a fast sequential atomic absorption spectrometer (280FS AA, Varian (Agilent Technology, Santa Clara, CA, USA)). The instrumental accuracy in FAAS analysis was appraised using certified reference material (CRM) 142 R and a good agreement for the 95% confidence interval was found, with recoveries in the range of 95–101%. Routinely, for FAAS analysis, each solution was analyzed in triplicate. In turn, Hg content (in plant or soil materials) was measured by AAS with a direct solids sampling using an advanced mercury spectrometer (AMA 254, Leco Corporation, St. Joseph, MI, USA). The instrumental accuracy in AAS with a direct solids sampling analysis was appraised using CRM SO-3. Consistently, for Hg content analysis, each solid material (soil, stems, roots) obtained from a single pot was analyzed in triplicate. In addition, for phytostabilized soils, a modified three-step sequential extraction was carried out in accordance with the European Community Bureau of Reference (BCR) procedure, elaborated by Pueyo, et al. [36]. The distribution of metal(-loid)s concentrations in discrete fractions extracted from phytostabilized soils, using sequential extraction and specific extractants, comprised FAAS measurement of four operationally defined fractions, differing in mobility: F1 (exchangeable and acid-soluble, mobile fraction; 0.11 M CH_3_COOH), F2 (reducible, potentially mobile fraction; 0.5 M NH_2_OH⋅HCl), F3 (oxidized, potentially mobile fraction; 30% H_2_O_2_/1 M CH_3_COONH_4_), and F4 (residual, stable fraction; aqua regia). After each extraction step, the resultant suspension was subjected to centrifugation and then filtered, thus obtaining an extract and a soil residue. At each step, an extract was collected into a PE bottle and stored at a temperature of 4 °C until FAAS analysis, while a soil residue was transferred to the next step and treated with appropriate extractant. The particle size distribution in initial soil was appraised with a laser diffraction particle size analyzer (Mastersize 2000, Malvern Panalytical, Malvern, Worcestershire, UK) and moisture content was measured as mass loss after heating samples at 105 °C using a moisture analyzer (MA X2. A, Radwag, Radom, Poland). The organic matter (OM) in soil materials (also in initial soil) was determined by soil combustion at 550 °C using a muffle furnace (ESM 9920, Carbolite, Sheffield, South Yorkshire, UK). The pH value of soil materials was measured in deionized water at a ratio of 1:2.5 *w*/*v*, in terms of suspension, using pH-meter (HI 221, Hanna Instrument, Woonsocket, RI, USA). Before setting up the vegetative experiment, biochar was milled in a cutting mill and additionally sifted using a 0.5 mm mesh. Its pH value was measured in deionized water at a ratio of 1:10 *w*/*v*, in terms of suspension. Nitrogen adsorption at 77 K was used to determine biochar specific surface area by the BET (Brunauer–Emmett–Teller) method using an accelerated surface area and porosity analyzer (ASAP 2020, Micromeritics, Norcross, GA, USA). The biochar was digested with the same reagents as soil materials in a microwave oven. The metal(-oid) content in biochar was measured by the same method as described above. Additionally, the scanning electron microscopy (SEM) images with energy-dispersive X-ray spectroscopy (EDS) of milled biochar particles (before and after freeze-thaw treatment) were taken using a scanning electron microscope (LEO 1430 VP, Jeol, Tokyo, Japan), with an acceleration voltage of 10 kV and 28 kV, respectively, for SEM images and EDS elemental composition of biochar surface.

### 2.4. Evaluation of Microbial Community Based on 16S rRNA Gene Amplicon Sequencing

Total genomic DNA was extracted from 500 µg of soil using a FastDNA SPIN Kit for Soil (MP Biomedicals) according to the manufacturer’s protocol. The DNA isolated from soil samples from each experimental replicate was mixed and the purity and content of the isolated DNA were measured using a NanoDrop solid spectrometer (Thermo Scientific, Waltham, MA, USA). The V4 hypervariable region of the 16S rRNA gene was amplified using the 515F/806R (5′-GTGCCAGCMGCCGCGGTAA-3′/5′-GGACTACHVGGGTWTCTAAT-3′) universal primer set [37] targeting bacterial and archaeal 16S rDNA genes. The amplicons were sequenced in the same run using the Illumina MiSeq platform at Research and Testing Laboratory (San Diego, CA, USA). Chimeras were removed from the raw reads by UCHIME [38] in de novo mode on the clustered, denoised data. The reads were condensed into FASTA format and removal of sequences with low-quality tags and a length less than half of the expected length was performed. Then the UPARSE algorithm [39] was used to cluster the obtained sequences into operational taxonomic units (OTUs). The centroid sequence from each cluster was run against a database of sequences from the NCBI using the USEARCH global alignment algorithm [40] to obtain taxonomic information. The raw sequencing data have been deposited in the NCBI Sequence Read Archive (SRA) as BioProjectPRJNA777436.

### 2.5. Statistical Analysis

Phytostabilization was conducted in three replications for each type of the soil mix (biochar 0% and 3%, *w*/*w*), under the following conditions: (1) greenhouse temperatures (before freeze–thaw) and (2) greenhouse temperatures, followed by FT chamber temperatures (after freeze–thaw treatment). In addition to this, analytical determinations were performed in three replicates for each material (soil, roots, and stems) obtained from a single pot, to demonstrate instrumental precision. The data obtained were statistically treated using the Statistica program version 13.3 for Windows. For data with significant differences identified by ANOVA, further analyses were conducted using Tukey’s HSD test (Statistica 13.1, TIBCO Software Inc., Palo Alto, CA, USA). When the ANOVA assumptions were not met, the Kruskal–Wallis test was used. For data with significant differences identified between variables, further analyses were conducted following the application of Tukey’s test (HSD). For statistical and meta-analysis of soil microbiome data, Microbiome Analyst [41,42] was used (*p* < 0.05). In complex microbial consortia, bacteria with a low abundance may be of great importance. Therefore, the number of reads was not normalized before calculation of diversity indices to maintain the highest possible precision for each soil sample [43].

## 3. Results and Discussion

### 3.1. The Effect of Biochar Amendment and Freeze-Thaw on Biomass and Chemical Composition of Lolium perenne L. Individual Organs

**Figure 1:**
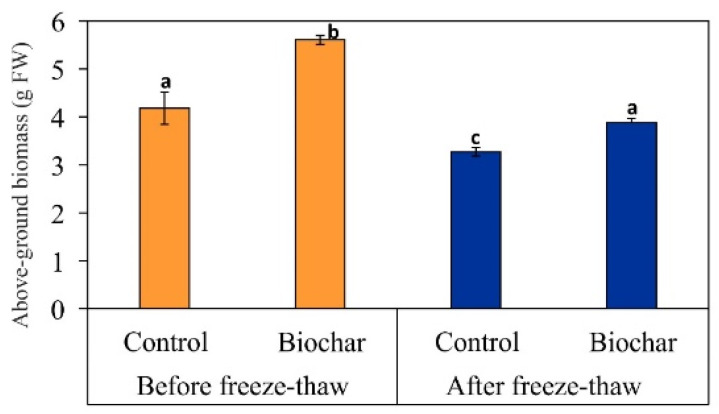
Effect of biochar amendment on above-ground biomass before and after freeze–thaw of a phytostabilized metal/metalloid-contaminated soil cultivated with *Lolium perenne* L. See Materials and Methods for detail on experimental set-up. Data are mean ± standard deviation (*n* = 3). Values followed by different letters differ significantly (ANOVA followed by Tukey’s HSD test, *p* < 0.05).

**Figure 2:**
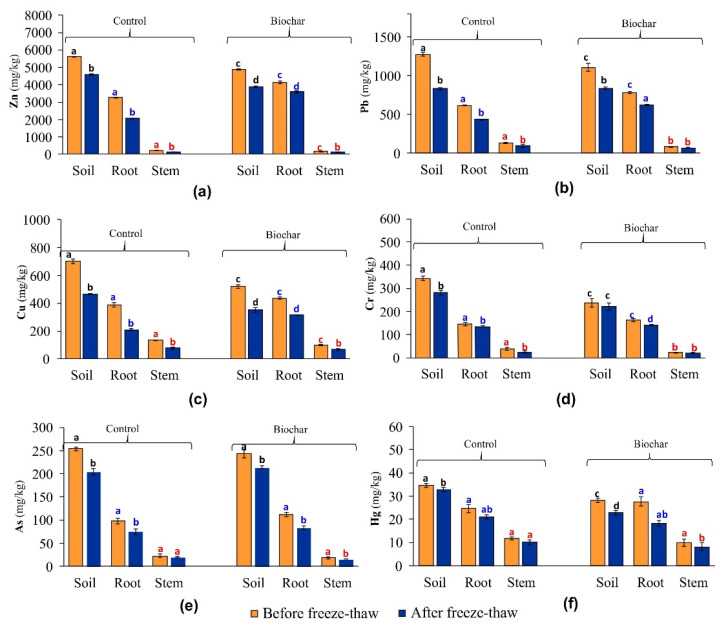
Effect of biochar amendment on distribution of selected metals/metalloids between soil and roots and stems of *Lolium perenne* L. before and after freeze–thaw treatment. The letters from (**a**–**f**) indicate the reading order of potentially toxic elements. Phytostabilization of the metal/metalloid-contaminated soil was as described in Materials and Methods. Data are mean ± standard deviation (*n* = 3). Different color letters: black for soil, blue for roots, and red for stems, indicate significant differences (*p* < 0.05).

The presence of potentially toxic metals/metalloids in the soil environment affects the growth and yield of plants. In such contaminated soil, the growth and crop quality, and biomass yield are, above all, dependent on the plant species, because there are differences in individual plants determined by their sensitivity to metals/metalloids and their cultivation requirements [44,45]. Thus, the ranges of toxicity of metals/metalloids vary depending on the element and individual plant species. The symptoms of plant diseases most often resulting from the presence of high content of metal(-loid)s in the soil include: yellowing of green parts (chlorosis), leaf, stem, or root necrosis, and a decreased rate and complete obstruction of their growth [46,47]. The climate and weather, which significantly influence the physiological processes occurring in plants, also cannot be ignored. The biochar effect and freeze–thaw impact on the biomass yield and metal(-loid) total content accumulating in plants (stems and roots) have been shown in Figure 1 and Figure 2, respectively. As seen from Figure 1, *Lolium perenne* L. above-ground biomass in both phytostabilized non-amended series (these are two control series, collected before and after freeze–thaw treatment, respectively) reveal higher sensitivity to contamination with metal(-loid)s occurring in soil than their corresponding biochar series, collected before and after freeze–thaw, respectively. As a consequence, a significantly lower biomass yield can be observed in each control series as compared to the corresponding biochar series. The recorded yield of above-ground biomass, in each phytostabilized biochar-amended series (collected before and after freeze–thaw, respectively), was, respectively, higher by 37% and 19%, as compared to the corresponding control series. Both of these results indicate that the addition of biochar to the soil significantly influences positive plant yield. Biochar leads to an increase in the content of Corg., P, N, Ca, Mg and K, and these elements improve soil fertility and thus crop quality and biomass yield [3]. A similar relationship was also confirmed by Gonzaga et al. [48], where biochar created from coconut husks and orange shells was applied as the soil amendment, significantly increasing the yield of *Brasica juncea* L. at the completion of phytostabilization conducted at moderate temperatures. On the other hand, a higher relative increase in biomass yield was obtained for the phytostabilized biochar-amended series before freeze–thaw (37%) versus a lower relative increase in above-ground biomass for the phytostabilized biochar-amended series after freeze–thaw treatment (19%), indicates that low temperatures, in terms of freeze–thaw conditions, act as a stronger factor influencing plant growth than the presence of additives in the soil (including biochar). It is known from the literature that, under conditions of low temperatures, damage to the cell walls of plants occurs on a large scale, and the denaturation of proteins and production of reactive oxygen species takes place, while ice formation in extracellular space leads to cell dehydration and the breaking of tissues [49]. Moreover, the increased frequency of the occurrence of freeze–thaw cycles also accelerates the loss of nutrients in the soil, (e.g., due to decomposition of compounds), which may lead to a lack of favorable conditions for plant growth [50]. The above facts help to elucidate why, despite biochar presence in soil, the freeze–thaw factor forces such an evident drop in the above-ground biomass yield, down to 3.8 g/pot (on the 116 d), as compared to 5.6 g/pot (on the 54 d) before undergoing freeze–thaw for younger counterparts.

Interaction between plant roots and soil nutrients always takes place, while its intensity depends on plant physiology, plant species, and soil chemistry. Large-scale aided-phytostabilization surveys indicate the presence of a higher accumulation of metal(-loid)s in plant roots than in their above-ground parts [15,51]. As seen from Figure 2 (bars for individual organs of *Lolium perenne* L.), a significantly higher accumulation of Zn, Pb, Cu, Cr, As, and Hg was found in plant roots than in the stems, both in non-amended and biochar-amended series, before freeze–thaw as well as after freeze–thaw treatment, respectively. This relationship was particularly visible following biochar addition to the soil. It is known from the available literature that at moderate temperatures, the addition of biochar leads to the immobilization of Cr, Zn, Pb, and Cd in soil, which, at the same time, leads to a decrease in the total content of these elements in above-ground parts of plants [52,53].

Interestingly, it may be noticed from Figure 2 (bars for *Lolium perenne* L. roots) that, in a phytostabilized biochar-amended series, a higher increase in the total content of selected metal(-loid)s in plant roots before freeze–thaw, was most visible in the following order: Pb (27%), Zn (26.6%), As (14%), Cu (13%), Cr (12%) and Hg (12%), as compared to the corresponding control series before freeze-thaw. However, in the phytostabilized biochar-amended series, after freeze–thaw, a higher increase in the total content of analyzed metal(-loid)s in roots was observed as follows: Zn (74%), Cu (50%), Pb (44%), As (11%) and Cr (7%), as compared to the corresponding control series after freeze–thaw. In contrast, the total Hg content was lower in roots in the biochar-amended series after freeze–thaw than in the corresponding control series after freeze–thaw. When applying sugarcane bagasse biochar to tannery polluted soils, Bashir, et al. [54] observed a significant decrease in Cr(VI) and Cr(III) content in *Zea mays* L. above-ground parts. Due to the fact that Zn is more easily assimilable by plants than Cd, its uptake by plants decreases in the presence of Zn [55]. Hart et al. [56] showed that Zn blocks the uptake of Cd by plants since both metals compete with each other during the transportation by a common carrier in the root cell membrane. Decreased uptake of As by plants in the presence of biochar can result from sorption of As by biochar particles or is connected with the increased soil pH value, which contributes to the precipitation of iron oxides in the rhizosphere [57,58].

### 3.2. The Effect of Biochar Amendment and Freeze-Thaw on Changes in Soil pH and Total Content of Metal(-loid)s in Soil

**Figure 3:**
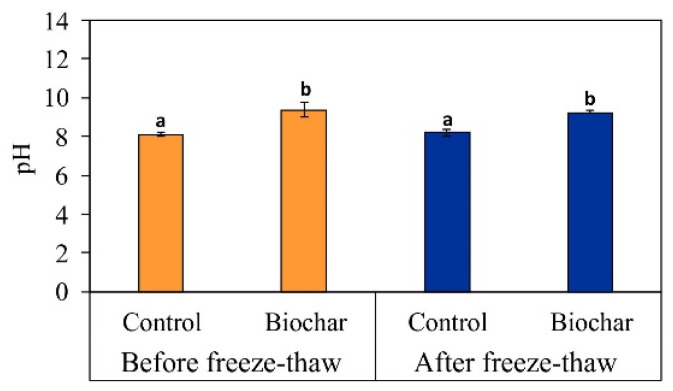
Effect of biochar amendment on soil pH value before and after freeze–thaw of a phytostabilized metal/metalloid-contaminated soil cultivated with *Lolium perenne* L. See Materials and Methods for detail on experimental set-up. Data are mean ± standard deviation (*n* = 3). Values followed by different letters differ significantly (ANOVA followed by Tukey’s HSD test, *p* < 0.05).

The soil pH value has an especially significant influence on the processes of initiating and immobilizing metal(-loid)s, both on farmland and in post-industrial areas [59]. As seen in Figure 3, there was no significant difference between before and after freeze–thaw treatment in terms of soil pH values. The application of biochar resulted in an increase in pH by 1.27 units (before freeze–thaw) and by 1.03 units (after freeze–thaw treatment) in relation to the corresponding control series before and after freeze–thaw treatment, respectively. Biochar is characterized by liming properties and thus contributes to decreasing exchangeable acidity and aluminum saturation in soil [60]. In studies by Yiang et al. [61], the addition of corn straw biochar to uncontaminated soil increased its pH by 0.10 units after thirty freeze–thaw cycles had been carried out.

**Figure 4:**
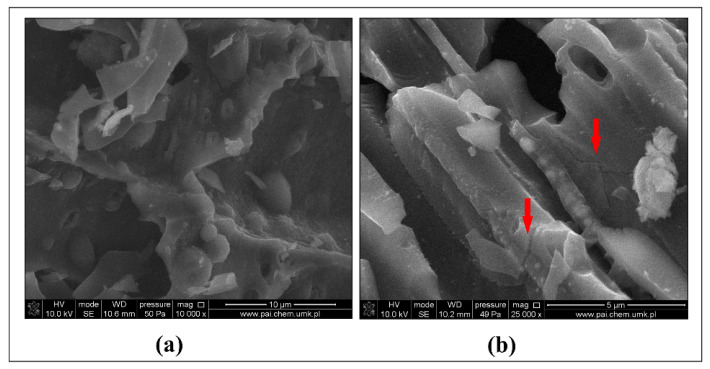
Scanning electron micrographs showing biochar before (**a**) and after (**b**) exposure to successive freeze-thaw cycles over 64 d. Red arrows indicate ‘cracks’ and ‘blunting’ of particle edges following freeze–thaw.

The immobilization of metal(-loids) in soil following the application of biochar takes place due to the calcification effect and adsorption, which may cover precipitation, complexing, cation exchange, and electrostatic attraction [62]. Metal(-loid)s occurring in soil compete for biding sites on biochar surface. Namgay et al. [57] showed that the sorption strength of metal(-loid)s by biochar decreases in the order of Pb > Cu > Cd > Zn > As. As seen from Figure 2 (bars for soil), the total content of Zn, Pb, Cu, Cr, As and Hg in the soil before exposure to freeze–thaw as well as after freeze–thaw treatment was significantly dependent on the addition of biochar. Its application, in phytostabilized soil before freeze–thaw, most significantly decreased the total content in the following order: Cr (32%), Cu (26%), Hg (19%), Pb (13%), Zn (12%), and As (4%) as compared to the corresponding control series before freeze–thaw. In phytostabilized biochar-amended soil after freeze–thaw, the observed decrease in the total content of the analyzed metal(-loid)s followed the respective order: Hg (31%), Cu (24%), Cr (21%), and Zn (15%) as compared to the corresponding control series after freeze–thaw. In contrast, a slight increase was observed for Pb (0.4%) and As (5.0%) in biochar-amended soil after freeze–thaw treatment. The decomposition of soil aggregates during the freeze–thaw cycles may have facilitated an increase in unstable metals [63] by releasing dissolved organic carbon, which makes metal(-loid)s more mobile [64]. The fact that multiple freezing–thawing cycles have a positive effect on the surface area of biochar is also of significance. Wang et al. [65] showed that, after exposure to 30 freeze–thaw cycles, the surface structure of biochar changed significantly in terms of increasing its surface area and pore structure. In our study, upon the completion of 16 freeze–thaw cycles (over 64 d), the surface area of biochar particles underwent an increase of nearly 2.1% when compared to particles that had not been subjected to such cycles. Before freeze–thaw treatment biochar was characterized by a surface area of 313.73 m²/g, which underwent an increase after 16 freeze–thaw cycles to a value of 321.01 m²/g. Coarse ground biochar is more susceptible to freeze–thaw in terms of creating additional cracks on its surface than fine biochar particles with a diameter equal to or less than 0.5 mm (herein under study) and, in consequence, the well-developed surface of biochar was found to have been only slightly enlarged after exposure to freeze–thaw. The small cracks resulting from exposure of biochar after 16 cycles were detected by additional SEM measurements and are shown as a vertical down arrow in Figure 4. Thus, following freeze–thaw, especially in the case of Hg and Cu occurring in soil, the content of these elements was lower with biochar application by 31% and 24%, respectively, when compared to the control series. Another outcome from the literature [35,65] is that, after exposure to multiple freeze–thaw, the number of functional groups containing oxygen (-OH, -COOH and -C=O) in biochar increases, which has a positive effect on the complexation of metals onto the biochar surface and their long-term immobilization in seasonally frozen soils. Thus, the resulting relative decrease in Hg and Cu content in the soil after exposure to freeze–thaw in our study can be assigned to the formation of functional groups rather than the observed slight increase in biochar surface area.

### 3.3. The Effect of Biochar Amendment and Freeze–Thaw on Metal(-loid)s Redistribution and Stability in Phytostabilized Soils

**Figure 5:**
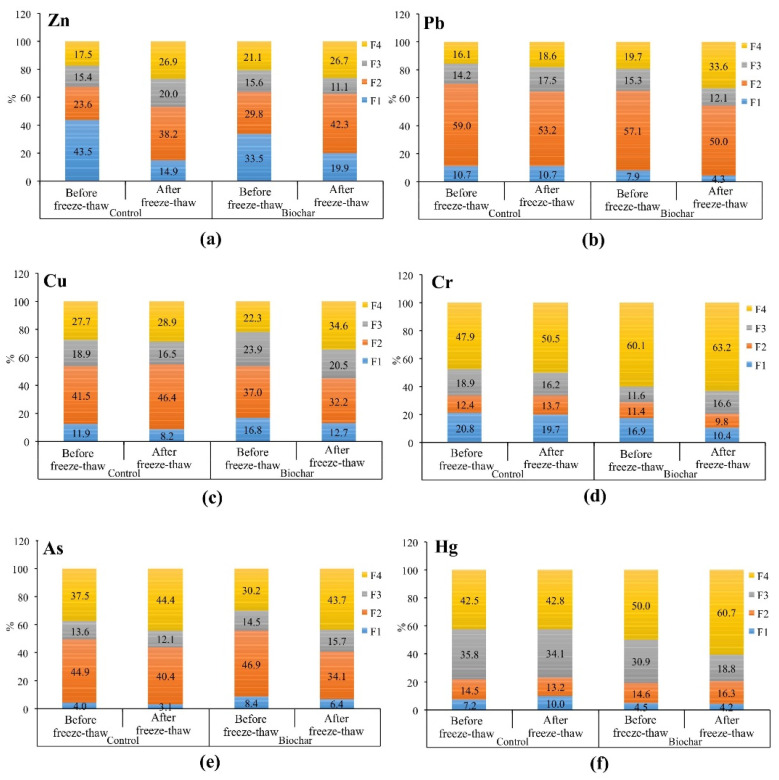
Changes in metal(-loid)s distribution (as percentage of individual fractions: F1—exchangeable and acid-soluble fraction, F2—reducible fraction, F3—oxidizable fraction, F4—residual fraction) in phytostabilized non-amended (control) and biochar-amended soil samples, before and after freeze-thaw. The letters from (**a**–**f**) indicate the reading order of potentially toxic elements. For an individual metal(-loid) and its specific fraction, values followed by different letters differ significantly in metal(-loid) share in the specific fraction between control and biochar-amended soils, before and after freeze–thaw (ANOVA followed by Tukey’s 401 HSD test, *p* < 0.05).

Application of biochar as a soil amendment and freeze–thaw affected metal(-loid)s distribution in phytostabilized soil (Figure 5). In phytostabilized control soil, before freeze–thaw, most of the metal(-loid)s, i.e., Pb, Zn, Cr, and Hg characterized by higher mobility (based on the share in the F1 fraction) compared to biochar-amended soil. This indicates a positive effect of biochar and phytostabilization process on metal(-loid)s immobilization. The most visible change in the metal(-loid)s mobility between amended and non-amended soil was observed for Zn, i.e., 43.5% of F1 (control soil) vs. 33.5% of F1 (biochar-amended soil). Biochar is known to enhance Zn transformations from mobile into stable fractions [66,67]. On the contrary, the share of F1 fraction for Cu and As was higher by a few percent in biochar-amended soil than in control. Biochar can increase the mobilization of some elements like Cu and As due to their complexation with dissolved organic matter from biochar [68]. An increase in As mobility by biochar can be also related to alkaline pH (higher in biochar-amended soil than in control soil) and possible redox reactions [68].

Among metal(-loid)s, Pb was the largest one associated with the oxides, followed by As and Cu. Arsenic in most soils exists mainly as oxyanions and exhibits a high affinity for Fe oxides [69]. Due to the presence of Fe oxides, biochar can improve metal redistribution into the F2 fraction [70]. In phytostabilized soil with biochar addition, only the shares of Zn and As in the F2 fraction were higher compared to control soil. This means that in biochar amended soil, metal(-loid)s were redistributed to other fractions than F2.

In phytostabilized soil with biochar, the shares of Cu, Pb, and As in oxidizable (F3) fraction were higher than in control soil. For Pb, Zn, Cr, and Hg there was an observed higher share in residual (F4) fraction in biochar-amended soil than in control soil. Among these elements, 60% of Cr and 50% of Hg were associated in the residual fraction. Mercury is the most toxic element in the environment. Its redistribution to a residual fraction under biochar treatment can be favorable in terms of decreasing environmental risk. Zhao et al. [71] found that Hg in polluted agricultural soil (total Hg 28.3 mg/kg, alkaline soil) prevailed in stable fractions, i.e., residual (bound to HgS) and organic (organo-complexed Hg), while water-soluble and exchangeable Hg fractions were much smaller. Despite different fractionation protocols of Hg used by Zhao et al. [71] and in our study, we also confirmed that Hg prevailed in stable fractions. It is known that Hg tends to bind to soil constituents involving clay, organic carbon, and sulfur [72]. Zhao et al. [71] found that biochar from risk husk containing sulfur can increase Hg sorption due to the strong binding affinity between sulfur and Hg, which forms the highly stable compounds. In this study, willow biochar was used as a soil amendment. Willow contains 0.04 wt% of sulfur, but its content can increase during higher temperatures of pyrolysis, up to 0.06 wt% at 400–550 °C, based on the elemental analysis (CHNS-O-ashes) performed using the TruSpec Micro instrument [73]. In this study, biochar was produced at 650 °C; thus, the sulfur content could be even higher. Herein, based on SEM/EDS measurement biochar contains 0.11 wt% of sulfur.

Application of 16 cycles of freeze–thaw had a visible effect on further transformations of metal(-loid)s, both in phytostabilized control soil and biochar-amended soil. Compared to control soil before freeze–thaw, in control soil after freeze–thaw the shares of metal(-loid)s in the F4 fraction and in the F2 fraction (except for Pb, Hg, and As) increased, and they decreased in F1 and F3 fractions (except for Pb and Hg in F1, and Pb and Zn in F3).

Although biochar caused visible changes in some fractions, (e.g., F4 fraction), freeze–thaw conditions themselves also affected redistribution of some metal(-loid)s from mobile to more stable fractions that were confirmed for control soil without amendment. However, freeze–thaw and biochar turned out to facilitate more metal(-loid)s redistribution into the most stable F4 fraction compared to control soil, by 5.8% for Cu, 15% for Pb, 12.7% for Cr, 17.9% for Hg. For Zn and As, their shares in the F4 fraction were comparable in control and amended soil after freeze–thaw. A degree of redistribution in F1-F3 fractions in biochar amended soil exposed to freeze–thaw depended on the type of metal(-loid). The share of all metal(-loid)s in the F1 fraction was decreased, to the greatest extent for Zn (by 13.7%). The share of the F2 fraction decreased for Cu, Pb, Cr, and As, but it increased for Zn and Hg. In the case of the F3 fraction, its share increased only for Cr and As.

Other authors confirm changes in metal(-loid) distribution because of freeze–thaw, and they vary depending on the type of metal(-loid) and matrix, (e.g., the solid phase of soil, sewage sludge). For example, Rui et al. [74] observed that after seven freeze–thaw cycles a visible redistribution of Cd and Pb occurred in soil, including an increase in the share of residual fraction, by 26.3–61.4% for Cd and by 67.4–80.3% for Pb. Yang et al. [75] found that wheat straw and corn straw biochars can stabilize Cd in alkaline soils under changing environmental conditions, including wet–dry and freeze–thaw. Wang et al. [76] observed that freeze–thaw improved Zn, Ni, Cu, Cr, Cd, and Pb stabilization in municipal sewage sludge blended with diatomite, FeSO_4_, and Ca(OH)_2_. Compared to the original sludge, the unstable fractions decreased, and the residual fractions of the metals increased. On the other hand, it was demonstrated that endogenous metals, (e.g., Cu, Cd) in biochar can be activated and their leachability can be increased due to dry–wet and freeze–thaw [77]. In the present study, in the soil–biochar–plant system, the mobility of metal(-loid)s after freeze–thaw was lower than before freeze–thaw. 

**Figure 6:**
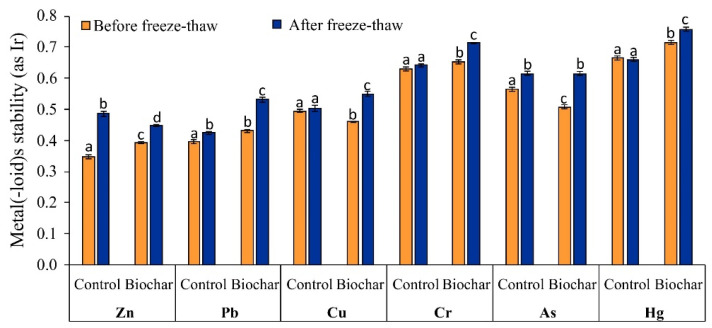
Changes in metal(-loid)s stability (as Ir) in phytostabilized non-amended (control) and biochar-amended soil samples, before and after freeze–thaw. For a given metal(-loid), different letters indicate significant differences in its stability in soil (*p* < 0.05).

Based on metal(-loid)s redistribution in soils, their stability was evaluated using the reduced partition index (Ir) (Figure 6). The Ir becomes a useful tool to evaluate metal-binding intensity in remediated soils. The proposed classification of metal stability based on the Ir can change as follows: lack of stability (Ir ≤ 0.1), low stability (0.1 < Ir ≤ 0.3), medium stability (0.3 < Ir ≤ 0.5), elevated stability (0.5 < Ir ≤ 0.7), high stability (0.7 < Ir ≤ 0.9) and very high stability (Ir > 0.9) [78].

In phytostabilized soil, before freeze–thaw, metal(-loid)s’ stability changed in the same order in control and in biochar-amended soil: Hg > Cr > As > Cu > Pb > Zn. However, the values of Ir for Hg (0.71), Cr (0.65), Pb (0.43), and Zn (0.39) in biochar-amended soil were higher (*p* < 0.05) than in control soil. After freeze–thaw, the stability of Cr, As, Pb, and Zn in control soil was higher (*p* < 0.05) than in control soil before freeze–thaw. In biochar-amended soil, the values of Ir for all metal(-loid)s were higher (*p* < 0.05) after freeze–thaw than before freeze–thaw.

The results on metal(-loid) stability in phytostabilized soil clearly indicate that biochar and freeze–thaw together facilitated more the increase (*p* < 0.05) in the stability of most metal(-loid)s than biochar or freeze–thaw alone. In phytostabilized soil amended with biochar after freeze–thaw, stability classification of Cu and Pb changed from medium to elevated, and for Cr from elevated to high. In phytostabilized control soil before freeze–thaw and after freeze–thaw, the stability of Cu, Pb, and Zn was classified as medium, and for Cr, Hg, and As as elevated. Despite a lack of change in the classification of metal(-loid)s’ stability in control soil, the values of Zn, Pb, and As were higher (*p* < 0.05) after freeze–thaw. This indicates that freeze–thaw causes physical and chemical changes both in soil and in biochar-amended soil, which in turn affected metal redistribution and their stability.

Freeze and thawing are the forms of biochar natural aging leading to the changes in its properties, such as physical fragmentation, reduction in the diameter of the particles, potential surface oxidation, and the release of dissolved organic matter or mineral dissolution [65,79]. Wang et al. [65] demonstrated that the number of freeze–thaw cycles is important for the changes in biochar properties. After 30 freeze–thaw cycles, the pH of corn straw biochar decreased by 0.81 unit, the surface area increased from 6.28 to 20.26 m^2^/g, the pore volume decreased from 0.009 mL/g to 0.003 mL/g, and pore diameter decreased from 1.692 to 1.423 nm. In the present study, both soil and biochar were alkaline, and the changes in soil pH after freeze–thaw were slight (pH increase by 0.08 unit in control soil and pH decrease by 0.17 unit in biochar-amended soil), thus carbonate, phosphate, or mineral phases were important for metals fixation through precipitation.

After freeze–thaw, the number of oxygen-containing functional groups in biochar can increase. As a result of these changes, the adsorption capacity of biochar after freeze–thaw cycles for Cu and Zn increased by 72 and 44%, respectively [65]. Freeze–thaw aging destroys the soil structure and consequently affects the soil’s capacity to sorbs metals [74]. Some changes in physicochemical properties of phytostabilized soils, with or without biochar addition, could occur in the present study. A decrease in freezing temperature increases the expansion of ice in soil, enhancing the extrusion strength of soil particles [80]. As a result, some changes in soil texture can be observed, which can improve metal sorption and redistribution. Due to the cracking of larger soil aggregates at freezing temperature, the specific surface area of soil particles increases which provides more ion adsorption sites, and enhances the cation exchange capacity [81]. Hou et al. [35] observed that after 16 freeze–thaw cycles, the soil fractions >2 mm were reduced from below 20% to 10.2% at −10 °C, to 8.7% at −20 °C and to 7.4% at −30 °C, while the content of silt and clay fractions increased. The presence of silt and clay fractions decides strong metal sorption in soil. In the present study, 16 freeze–thaw cycles (−20 °C/+20 °C) were used for phytostabilized soil. The changes in soil texture after freeze–thaw can be one of the potential reasons explaining an increase in the stability of some metal(-loid)s in control soil in this study.

### 3.4. Influence of Biochar Amendment and Freeze-Thaw on Microbial Community in Soil

The composition of the microbial community in soil samples was characterized using a 16S rRNA gene amplicon sequencing procedure, which resulted in over 155 thousand high-quality reads with an average of 38947 ± 3367 reads per sample (Table 1). The amount of extracted, high-quality DNA averaged 321 ± 23 ng/µL. Sequencing depth was sufficient as assessed based on rarefaction curves (Figure S1) and the number of observed operational taxonomic units OTUs was about 1900 and about 1600 for soil samples before and after freeze–thaw, respectively. Chao1 index, informing about species richness based on their abundance, varied from 1783.8 to 2132.2. Both Chao1 and the total number of OTUs were significantly higher in unfrozen samples than in samples subjected to freeze–thaw (*t*-test, *p* = 0.01).

Analysis of the dendrogram constructed based on the metagenomic profile of each soil sample indicated that freeze–thaw, not biochar addition, determined the microbial composition of the soil (Figure S2). Biochar addition, however, relieved the effects of freeze–thaw stress because it positively affected microbial diversity in soil (Table 1). In samples subjected to freeze–thaw, the Shannon index of diversity decreased by about 8% for control soil and only about 4% in biochar-amended soil. Highly-porous biochar provides a habitat for microorganisms, protects them from predators, and increases microbial biomass [82,83]. As a result, a diverse microbial community able to conduct a wide range of metabolic traits can support plant growth in trace element contaminated soils by enhancing retention of potentially toxic elements in roots and helping plants to acquire nutrients and recycle the organic matter [84]. The principal component analysis (PCA) depicting beta diversity between the analyzed soil samples (data not shown) has shown the first two principal components explained over 97% of the total variance of data.

Many microbes developed metal resistance mechanisms that help them to reduce metal toxicity [85,86]. Metal(-loid)s species can be extruded outside the microbial cell surface, adsorbed onto the cell wall, bioaccumulated inside cells, or biotransformed to less toxic forms [87,88,89]. Bacteria, which do not possess such mechanisms of resistance to metal(-loid)s, will be eliminated from a soil community, which results in lowering the richness and evenness of bacterial species and thus stability and ecological balance. This negative effect of metal(-loid)s can be harsher if the soil community is additionally subjected to temperature changes, especially freezing. Freezing lyses microbial cells and releases their nutrients into soils but the data regarding the freeze–thaw effect on soil community is unclear. Some reports indicate that freezing increases microbial activity [90], while others state otherwise [91]. Negative effects of both metal(-loid)s presence and soil freezing can be reduced by soil amendments. It was reported that biochar amendments increase soil nutrient content, activity, and diversity of the microbial community in trace element-exposed soils and thus increase soil health [15,92].

**Figure 7:**
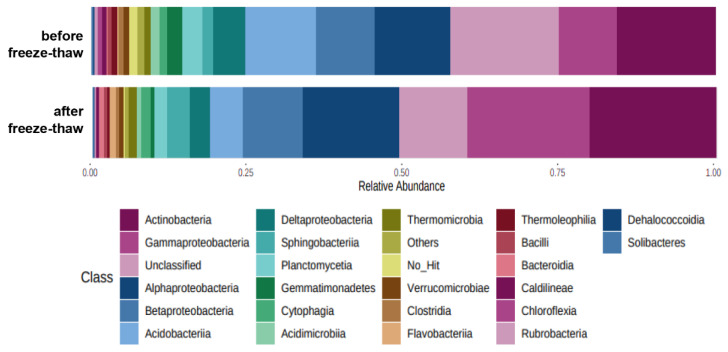
A stacked bar graph presenting microbial composition at a class level of soil samples, (relative percentage abundance of particular sequences among all obtained sequences), 25 most abundant taxa presented, samples were merged to groups by freeze–thaw after completed phytostabilization under the following conditions: (1) greenhouse temperatures (before freeze–thaw) and (2) greenhouse temperatures, followed by FT-chamber temperatures (after freeze–thaw).

At a class level, freeze–thaw strongly increased the abundance of *Actinobacteria*, *Sphingobacteria*, *Flavobacteria* and *Rubrobacteria* in soil (Figure 7); the abundance of *Rubrobacteria* was significantly higher than in soils before freeze–thaw. Those classes usually predominate in soil and in the rhizosphere of metallophytes, significantly affecting phytoremediation effectiveness [93,94]. The metallophytes as a specific plant can tolerate high levels of potentially toxic elements. Freeze–thaw affected the distribution of classes within phyla *Proteobacteria*. In freeze–thaw-exposed samples, abundances of *Gammmaproteobacteria* and *Alphaprotepbacteria* significantly increased, the abundance of *Betaproteobacteria* did not change and the average abundance of Deltaproteobacteria decreased. A study by Navas et al. [95] has shown that *Proteobacteria* predominated in soil with a high content of metal(-loid)s while *Actinobacteria* were more abundant in soil with low content of metal(-loid)s. The higher resilience of *Proteobacteria* to high content of metal(-loid)s can be related to the fact that they are Gram-negative bacteria and possess an outer lipopolysaccharide layer in their cell wall that sequestrates metals extracellularly conferring adaptation to harsh contaminated environments [96]. *Actinobacteria* are an important member of soil microbiome participating in carbon cycling and production of soil organic matter [94,97]. The good ability of *Actinobacteria* to maintain their metabolic activities under low temperatures can be explained by cross-feeding or cooperation with other soil bacterial species [98].

Freeze–thaw decreased abundances of *Acidobacteria*, *Planctomycetia* and *Gemmatimonadetes* (Figure 7). This observation is in contrast with previous studies showing that *Acidobacteria* and *Gemmatimonadetes* comprised predominant classes in soils from polar regions [99] and application of freeze–thaw at different amplitudes in uncontaminated soils increased their abundances [100].

**Figure 8:**
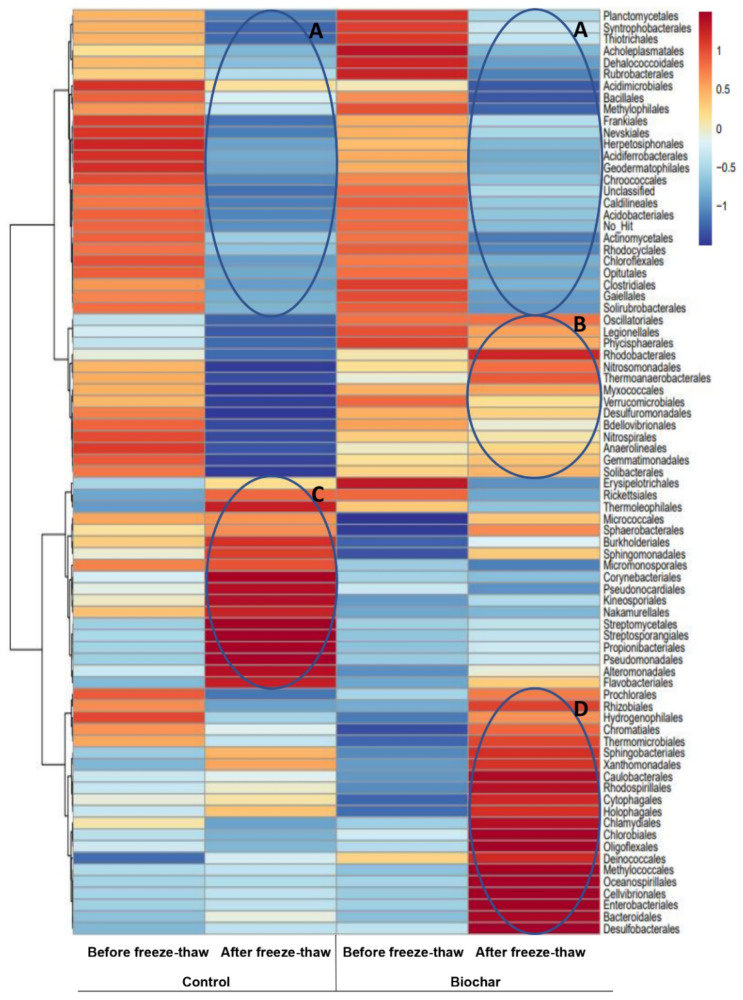
Heatmap presenting differences in relative abundance of particular sequences among all obtained sequneces between phytostabilized non−amended (control) and biochar−amended soil samples, respectively, (order level of microbial composition, Euclidean distance measure, Ward algorithm used for clustering); A, B, C, D indicate particular groups of microorganisms, for explanation see the text. Phytostabilization was carried out under the following conditions: (1) greenhouse temperatures (before freeze−thaw) and (2) greenhouse temperatures, followed by FT chamber temperatures (after freeze−thaw).

The addition of biochar in soil stimulated the growth of bacteria belonging to orders *Oscillatoriales*, *Legionellales*, *Physicispherales,* and in soil amended with biochar but not subjected to freeze–thaw also strongly increased abundances of *Erysipelotrichales* and *Acholeplasmatales* (Figure 8). Erysipelotrichales abundance is favored by the high availability of nutrients, which was ensured by the addition of biochar. In a study by Bhardwaj et al. [101], order *Erysipelotrichales* was exclusively present in mesocosms biostimulated by using a mustard seed meal nitrogen-rich supplement. The mesocosms, or experimental enclosures, are designed to provide a limited body of soil with close to natural conditions, in which environmental factors can be realistically manipulated. Mhete et al. [94] observed that in samples from garden soil, saline soil, and sludge-impacted soil, *Erysipelotrichales* (7%) was present only in sludge-impacted soil characterized by higher P, Ca, and carbon content. The presence of biochar in soil not subjected to freeze–thaw negatively affected the growth of mostly *Micrococcales*, *Sphaerobacterales,* and *Chromatiales* (Figure 8).

Application of freeze–thaw visibly decreased abundances of *Planctomycetales*, *Syntrophobacterales*, *Thiotrichales,* and *Frankiales* in soil non-amended with biochar and *Rubrobacterales*, *Acidimicrobiales*, *Bacillales,* and *Methylophilales* in biochar-amended soil (Figure 8, group A). However, the addition of biochar to soil released the negative effect of freeze–thaw for a group of bacterial orders (Figure 8, group B) crucial for chemical conversions in soil, especially for N cycling. In general, freeze–thaw increases inorganic nitrogen losses through leaching from the soil, and as a result, a decrease in the biomass of the crop is observed the following growing season [102]. In our study, this N leaching may have resulted in a significant decrease in the abundance of both *Nitrosomonadales* and *Nitrospirales* that was observed in the unamended control soil. Biochar amendment increased soil N retention during freeze–thaw and N accessibility to microorganisms thus favoring the growth of *Nitrosomonadales* and *Nitrospirales*. Biochar addition also allowed the preservation of *Verrucomicrobia*, *Gemmatomonadales,* and *Thermoanaerobacterales*. Microorganisms belonging to *Verrucomicrobia* prefer high soil pH and total carbon and nitrogen content [103], ensured by biochar addition, and take part in both nitrogen fixation [104] and polysaccharide degradation in soils [105]. Gemmatomonadales have a unique regulatory mechanism of N_2_O reduction [106] while members of *Thermoanaerobacterales* can metabolize recalcitrant organic compounds at high N content in soils [101,107].

Freeze–thaw increased the relative abundances of sequences belonging to *Sphingomonadales*, *Sphingobacteriales*, *Xanthomonadales,* and *Sphaerobacterales* in the overall amount of identified sequences. Those orders contain such genera as *Sphingobium*, *Lysobacter*, *Sphingomonas,* or *Pseudoxanthomonas* which are commonly identified in soils contaminated with potentially toxic elements and are involved in their transformations [93]. Moreover, bacteria belonging to those orders, cope well with harsh environmental conditions, e.g., high salinity [94,108] which make them desirable members of the community because their metabolism can gradually improve soil properties and create conditions for the growth of more demanding microorganisms.

Some bacterial orders coped well with low temperatures (freeze–thaw applied) but preferred a low-nutrient environment (no biochar addition) (Figure 8, group C). Most bacteria belonging to those orders were identified in environments characterized by low temperatures [75] and possess metal resistance genes [109,110]. Within this group, *Pseudomonadales* are one of the best-recognized orders in trace element contaminated soils. An increase in the abundance of *Pseudomonadales* is desirable because microorganisms from this order turned out to be an efficient biosorbent for the removal of Cd and other potentially toxic elements from solution, contaminated waste, water, and soil [111]. Their growth in the soil also promotes rhizobacteria activity and biosurfactant production, which plays a key role in metal phytoextraction processes [112].

Finally, a group of bacterial orders was distinguished that coped well in biochar-amended soil subjected to freeze–thaw (Figure 8, group D). Within this group, many orders such as *Rhizobialesor*, *Rhodospirillales* are recognized as important components of soil microbiota, independent of soil properties [94]. *Rhizobiales* have a good tolerance to potentially toxic elements and were indicated as predominant species in soil contaminated with Cd [93] or Pb [113]. Some methanotrophs belonging to *Rhizobiales* possess genes necessary for the reduction of Hg(II) and As(V) and can mitigate the toxicity of potentially toxic elements [114]. *Rhizobiales* are well-known beneficial partners in plant-microbe interactions providing various nutrients, phytohormons as well as precursors for essential plant metabolites for their hosts [115]. The order contains many genera of N-fixing, microsymbiotic bacteria that have a key role in the adaptation of legumes to soils poor in nitrogen and with high content of metals [116]. In this group, several bacterial orders contributing to the sulfur cycle were also identified. For example, *Chlorobiales* use sulfide ions as electron donors to produce elemental sulfur [117] while *Desulfobacterales* conduct the dissimilatory sulfate reduction, in which sulfur compounds could be utilized as potential energy sources for microorganisms [118]. Many of the identified orders such as *Rhodospirillales*, *Oceanospirillales*, *Bacteroidales,* or *Chlorobiales* [119] were previously identified as preferring nutrient-rich environments, which may explain their abundance in biochar-amended soil.

**Figure 9:**
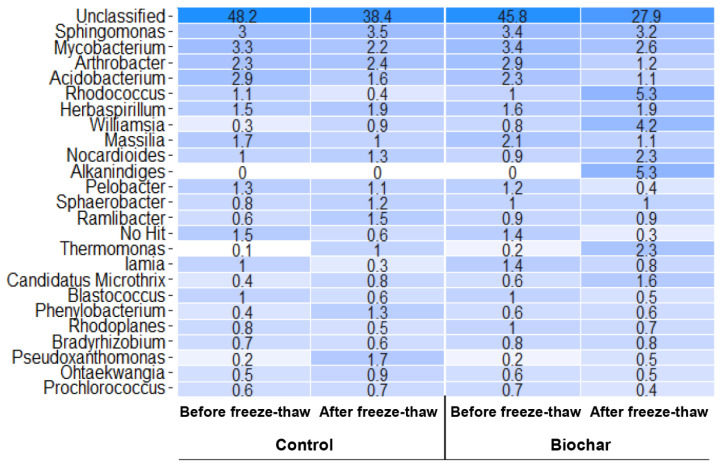
Heatmap of the genera with the highest percentage abundances in phytostabilized non-amended (control) and biochar-amended soil samples, respectively. Phytostabilization was carried out under the following conditions: (1) greenhouse temperatures (before freeze–thaw) and (2) greenhouse temperatures, followed by FT-chamber temperatures (after freeze–thaw).

The most numerous classified bacterial genera are presented in Figure 9. *Sphingomonas* sp. and Mycobacterium sp. were present in all samples with abundances at a level of 3 and 2–3%, respectively. Both genera were reported in soils contaminated with potentially toxic elements. Navas, et al. [95] identified *Mycobacterium* sp. as a predominant genus in soil from mining sites but with a low content of potentially toxic elements while *Sphingomonas* sp. was abundant in soil contaminated with Ni^2+^ [93]. Bacteria from both genera possess trace element resistance genes [120]. *Sphingomonas* sp. contains copper resistance genes in plasmids that contribute to the distribution of these genes in the soil bacterial community increasing the resistance of the microbiome to trace element pollution [120]. Freeze-thaw significantly increased the abundance of *Thermomonas* sp. which was favorable because this genus showed high tolerance to soil pollution with Ni^2+^, Co^2+^, and Cd^2+^, and was perceived as a potential source of microorganisms useful for bioaugmentation of soils contaminated with these potentially toxic elements [93]. In control soil subjected to freeze–thaw high abundance of *Pseudoxanthomonas* sp. was observed (1.7%). *Pseudoxanthomonas* sp. is important in the soil environment due to the ability to degrade nitrite, nitrate, and different hydrocarbons [121]. This capacity might help them to adapt well to both low temperatures and low levels of nutrients in the environment. It was reported that *Pseudoxanthomonas* sp. may remove Hg from soils [122] and their biosurfactants can be used for bioremediation of hydrocarbon-contaminated soils [123].

In biochar-amended soil after freeze–thaw, *Rhodococcus* sp. (5.3%), *Williamsia* sp. (4.3%) and *Alkalindiges* sp. (5.3%) were most abundant; abundances of *Williamsia* sp. and *Alkalindiges* sp. were significantly higher than is soils before freeze–thaw. Both *Rhodococcus* sp. and *Williamsia* sp. are psychrotolerant genera that are common in low-temperature areas such as the Antarctic [124,125]. The genomic features of *Williamsia* sp. isolated from the rhizosphere indicate their putative adaptions to UV radiation, heat and cold shock, desiccation, and oxidative stress [126]. *Rhodococcus* sp. is, on the other hand, reported, next to, e.g., *Bacillus* or *Pseudomonas* sp., as a genus with the highest resistance against potentially toxic elements, especially as [89].

## 4. Conclusions

The effects of lowered temperature are accompanied by both, physical and physiological changes in plants, but also significantly influence the chemical and microbiological properties of soil. Biochar contributed to an increase in *Lolium perenne* L. yield, with a similar relationship observed following freeze–thaw, though in this case, the crop yield was significantly lower. Biochar-aided phytostabilization before freeze–thaw resulted in a higher total content of Pb, Zn, As, Cu, and a similar level of Cr and Hg in *Lolium perenne* L. roots. However, biochar-aided phytostabilization after freeze–thaw caused the occurrence of a higher content of Zn, Cu, Pb, As, and Cr in plant roots. Upon adding biochar to soil and prior to setting up exposure to freeze–thaw, the total contents of Cr, Cu, and Hg in soil were most reduced as compared to the control series. Following freeze–thaw cycles, especially in the case of Hg, Cu, and Cr following the application of biochar, the content of these elements was lower in relation to the control series. Biochar and freeze–thaw cycles facilitated an increase in the stability of most metal(-loid)s more than biochar or freeze–thaw alone, especially for Cu, Pb, Hg, and Cr. The increase in metal(-loid) stability resulted from their redistribution mainly to a residual fraction. Freeze–thaw caused visible changes in metal(-loid) redistribution and their stability in phytostabilized soils. The microbial composition of the soil microbiome was determined by freeze–thaw. The core genera in soil, resistant to freeze–thaw, were *Sphingomonas* sp. and Mycobacterium sp. The application of biochar decreased the negative effect of freeze–thaw on bacterial diversity and helped to preserve bacterial groups important for efficient nutrient conversions in soil, such as *Nitrosomonadales*, *Nitrospirales,* or *Verrucomicrobia*. Freeze–thaw increased the abundance of *Thermomonas* sp. and in biochar-amended soil exposed to freeze–thaw, *Rhodococcus* sp., *Williamsia* sp., and *Alkalindiges* sp. were most abundant. Knowledge regarding the influence of low temperatures on the course of processes of aided phytostabilization can prove to be especially useful when planning the implementation of this technique in areas with changeable temperature conditions.

## Figures and Tables

**Table 1:** Alpha diversity (within sample diversity) values for phytostabilized non-amended (control) and biochar-amended soil samples calculated based on the amplicon analysis. Phytostabilization was carried out under the following conditions: (1) greenhouse temperatures (before freeze-thaw) and (2) greenhouse temperatures, followed by FT-chamber temperatures (after freeze-thaw).

Sample		Reads	Observed OTUs	Shannon Index of Diversity	Chao1 Index
Control	before freeze–thaw (1)	41,087	1938	5.99	2132.2
	after freeze–thaw (2)	41,914	1600	5.49	1783.8
Biochar	before freeze–thaw (1)	34,445	1875	6.12	2086.1
	after freeze–thaw (2)	38,343	1673	5.85	1850.6

## Data Availability

Not applicable.

## Supplementary Materials

The following supporting information can be downloaded at: https://www.mdpi.com/article/10.3390/ma15113801/s1, Figure S1: Rarefaction curves obtained for the analyzed soil samples.; Figure S2: A dendrogram presenting similarities of microbial communities between the analyzed samples (genus level, distance measured based on Bray–Curtis index, Ward algorithm used for clustering).
