# Supplementary material for: Effect of Biochar on Metal Distribution and Microbiome Dynamic of a Phytostabilized Metalloid-Contaminated Soil Following Freeze–Thaw Cycles
Source: Materials (Basel). 2022 May 26;15(11):3801. doi: 10.3390/ma15113801 (PMC9181493; doi:10.3390/ma15113801)
Supplement: Supplementary file 1 [file materials-15-03801-s001.zip › materials-1689910-supplementary.pdf]

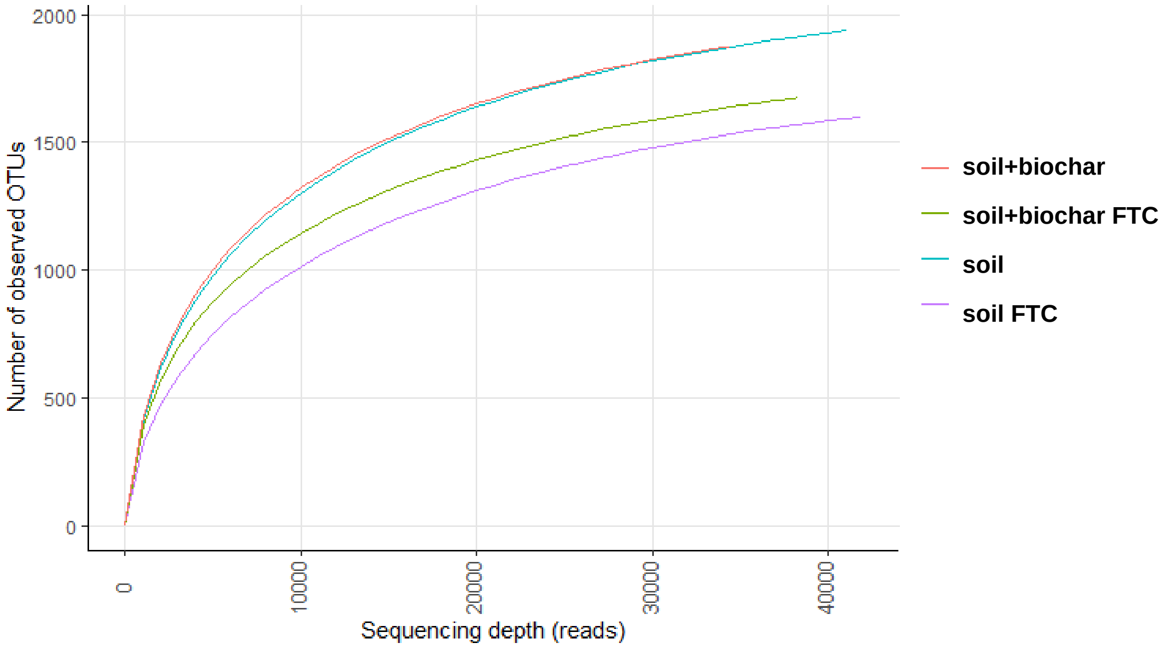

Figure S1. Rarefaction curves obtained for the analyzed soil samples.

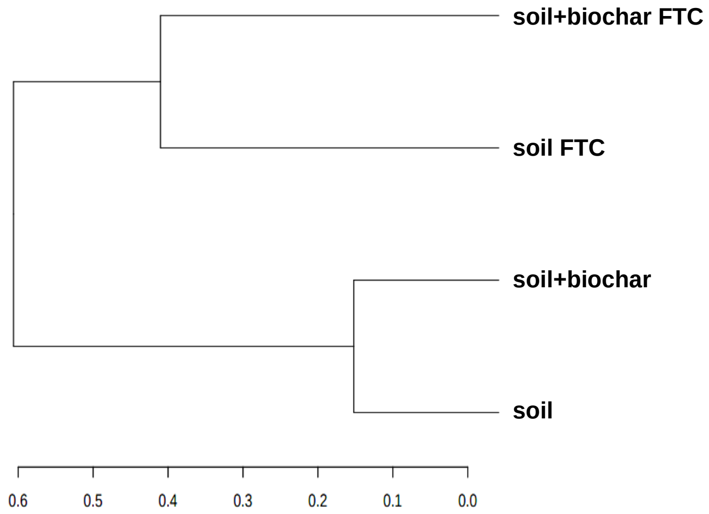

Figure S2. A dendrogram presenting similarities of microbial communities between the analyzed samples (genus level, distance measured based on Bray-Curtis index, Ward algorithm used for clustering).
